# *In vivo* Retinal Fluorescence Imaging With Curcumin in an Alzheimer Mouse Model

**DOI:** 10.3389/fnins.2020.00713

**Published:** 2020-07-03

**Authors:** Ahmad Sidiqi, Daniel Wahl, Sieun Lee, Da Ma, Elliott To, Jing Cui, Eleanor To, Mirza Faisal Beg, Marinko Sarunic, Joanne A. Matsubara

**Affiliations:** ^1^Department of Ophthalmology & Visual Sciences, University of British Columbia, Vancouver, BC, Canada; ^2^School of Engineering Science, Simon Fraser University, Burnaby, BC, Canada

**Keywords:** amyloid beta, plaques, Alzheimer’s Disease, APP/PS1, fluorescence, scanning laser ophthalmoscopy, retinal ganglion cell

## Abstract

Alzheimer’s disease (AD) is characterized by amyloid beta (Aβ) plaques in the brain detectable by highly invasive *in vivo* brain imaging or in post-mortem tissues. A non-invasive and inexpensive screening method is needed for early diagnosis of asymptomatic AD patients. The shared developmental origin and similarities with the brain make the retina a suitable surrogate tissue to assess Aβ load in AD. Using curcumin, a FluoroProbe that binds to Aβ, we labeled and measured the retinal fluorescence *in vivo* and compared with the immunohistochemical measurements of the brain and retinal Aβ load in the APP/PS1 mouse model. *In vivo* retinal images were acquired every 2 months using custom fluorescence scanning laser ophthalmoscopy (fSLO) after tail vein injections of curcumin in individual mice followed longitudinally from ages 5 to 19 months. At the same time points, 1–2 mice from the same cohort were sacrificed and immunohistochemistry was performed on their brain and retinal tissues. Results demonstrated cortical and retinal Aβ immunoreactivity were significantly greater in Tg than WT groups. Age-related increase in retinal Aβ immunoreactivity was greater in Tg than WT groups. Retinal Aβ immunoreactivity was present in the inner retinal layers and consisted of small speck-like extracellular deposits and intracellular labeling in the cytoplasm of a subset of retinal ganglion cells. *In vivo* retinal fluorescence with curcumin injection was significantly greater in older mice (11–19 months) than younger mice (5–9 months) in both Tg and WT groups. *In vivo* retinal fluorescence with curcumin injection was significantly greater in Tg than WT in older mice (ages 11–19 months). Finally, and most importantly, the correlation between *in vivo* retinal fluorescence with curcumin injection and Aβ immunoreactivity in the cortex was stronger in Tg compared to WT groups. Our data reveal that retina and brain of APP/PS1 Tg mice increasingly express Aβ with age. *In vivo* retinal fluorescence with curcumin correlated strongly with cortical Aβ immunohistochemistry in Tg mice. These findings suggest that using *in vivo* fSLO imaging of AD-susceptible retina may be a useful, non-invasive method of detecting Aβ in the retina as a surrogate indicator of Aβ load in the brain.

## Introduction

Alzheimer’s disease (AD) is a chronic irreversible neurodegenerative disease that leads to progressive memory loss and cognitive impairment. With the aging population, the burden of AD is increasing. A recent study estimates that 1 in 5 adults past the age of 85 will be affected, and AD will afflict over 100 million people worldwide by the year 2050 (Brookmeyer et al., 2007). The only method for confirmed AD diagnosis is post-mortem histological evidence of cerebral amyloid beta (Aβ) aggregates in the form of senile neuritic plaques and intraneuronal neurofibrillary tangles composed of hyperphosphorylated tau (Rowan et al., 2007; Sorrentino and Bonavita, 2007). In patients, extensive neurocognitive assessment, along with laboratory tests and brain imaging are used to diagnose dementia of Alzheimer’s type. While no cure presently exists, early diagnosis of AD can lead to treatment that helps to manage the symptoms, maximize functionality, and maintain quality of life (Sevigny et al., 2016). The major challenge for early AD diagnosis is the identification of biomarkers by non-invasive methods that could be readily deployable to the at-risk population. This goal is further challenged because the exact pathophysiology and corresponding onset of the disease remain poorly understood.

The amyloid cascade hypothesis remains the most widely accepted mechanism of the AD pathogenesis. It posits that Aβ 1-42 accumulation, via post-translational proteolytic cleavage of amyloid beta peptides (APP), precedes aggregation into senile plaques and subsequent neuronal damage (Beyreuther and Masters, 1991; Hardy and Allsop, 1991; Selkoe, 1991; Hardy and Higgins, 1992). Whether this Aβ accumulation is due to overproduction or decreased clearance, or a product of both, remains unknown. Positron emission tomography (PET) and cerebral spinal fluid (CSF) analysis are validated methods of measuring Aβ as a means of predicting and diagnosing AD in patients (Rabinovici et al., 2011; Ferreira et al., 2014). However, both techniques have drawbacks that limit their clinical use for patients with AD or at risk of AD. PET imaging exposes patients to ionizing radiation, requires expensive equipment, and has low spatial resolution. CSF analysis has limited sensitivity and specificity and requires invasive procedures for sample collection (Bateman et al., 2012; Fagan et al., 2014). Both techniques detect late manifestations of AD, such as brain atrophy, Aβ and phosphorylated tau accumulation – signs of advanced and potentially irreversible neuronal injury. Identifying a biomarker that is upstream to this damage using inexpensive and less invasive techniques is desired, as it may provide both a wider therapeutic potential and a novel method of detecting and monitoring disease progression.

AD patients have higher rates of neurovisual impairments compared to their age-matched controls (Schlotterer et al., 1984; Sadun et al., 1987; Cronin-Golomb et al., 1993; Risacher et al., 2013; Chang et al., 2014; Tzekov and Mullan, 2014). This, along with the evidence that Aβ plaques accumulate in the visual cortex prior to depositing in the hippocampal area (McKee et al., 2006) prompted research into ocular biomarkers. The retina is an extension of the central nervous system that is uniquely visible through the eye’s path of light, making it an ideal structure for optical imaging. Given that it shares its embryological origin with the brain, the retina has been extensively investigated as a region that may mirror AD changes in the brain, and is impacted in AD patients (Bayhan et al., 2015; Hart et al., 2016). Optical coherence tomography (OCT) is a common retinal imaging modality that has been explored for non-invasive diagnosis of AD (Kesler et al., 2011; Coppola et al., 2015). However, macular and peripapillary retinal fiber layer thickness abnormalities observed in AD by OCT are limited in their utility for AD-specific screening due to their similarities with abnormalities observed in other systemic diseases and retinal disorders (Kergoat et al., 2001; Doustar et al., 2017).

Post-mortem analysis of the eyes from human donors and the APP/PS1 and other mouse models of AD, which mimics human AD pathology through overexpression of APP and deposition of Aβ brain plaques, have shown APP and/or Aβ accumulation in the retina (Ning et al., 2008; Shimazawa et al., 2008; Dutescu et al., 2009; Liu et al., 2009; Perez et al., 2009; Kam et al., 2010; Park et al., 2014; Gupta et al., 2016; Hadoux et al., 2019). More recently, Koronyo-Hamaoui et al. (2011) used tail-vein injections of curcumin, a natural FluoroProbe that specifically binds to the beta sheet formation of Aβ, in APP/PS1 mouse and found that retinal Aβ deposits precede brain plaque formations (Koronyo et al., 2012). However, one question that remains unanswered in the current literature is whether it is possible to estimate the degree of Aβ deposition in the brain by measuring Aβ in the retina. If this hypothesis stands, the retina could be a surrogate tissue to assess Aβ deposition in the brain.

In this study, we quantified the retinal expression of Aβ by comparing *in vivo* curcumin fluorescence obtained from fluorescence scanning laser ophthalmoscopy (fSLO) with retinal and brain immunohistological labeling of Aβ in the APP/PS1 mouse model of AD in order to compare the brain and eye deposition of Aβ in young and old APP/PS1 transgenic (Tg) and wild-type (WT) mice and investigate the potential of *in vivo* retinal imaging with curcumin labeling as a surrogate method for quantifying Aβ load in the brain.

## Materials and Methods

### Animals

The mouse imaging was performed under the protocols approved by the University Animal Care Committees at the University of British Columbia and Simon Fraser University, conformed to the guidelines of the Canadian Council on Animal Care, and in accordance with the Resolution on the Use of Animals in Research of the Association of Research in Vision and Ophthalmology. APP/PS1 Tg mice (*N* = 12, JAX stock #004462) and their non-Tg sibling WT (*N* = 11) controls were purchased at eight-weeks of age. The mice were raised using standard care and chow. Two of each Tg and WT mice died before the first imaging session, reducing the number of animals to 10 Tg and 9 WT mice. From 5-months of age, mice were imaged every 2–3 months until 19-month old at **7** time points. Starting from 5-month old, 1–2 mice from the Tg and WT cohort were euthanized after imaging and their ocular and brain tissue were preserved for immunohistological processing. In addition to these animals, several Tg mice (*N* = 3) and WT (*N* = 3), aged 6–18 months, were sacrificed to assess amyloid beta deposits in the retina and brain without the curcumin injections or *in vivo* imaging.

### *Ex vivo* Cortical and Retinal Cross-Section Aβ Immunohistochemistry

Frozen 8 μm coronal brain tissue sections from Tg and WT mice were cut on a cryostat and mounted on glass slides. Paraffin embedded eye tissues from Tg and WT mice (sacrificed at 6, 9, or 18 months) were cut at 6 μm thickness. All sections underwent antigen retrieval in 88% formic acid for 5 min at room temperature and were washed three times for 5 min with PBS (pH 7.4). A subset of brain and eye sections underwent chromogenic visualization of Aβ immunohistochemistry and were then treated with 1% hydrogen peroxide in distilled water for 15 min at room temperature to eliminate endogenous peroxidase activity and washed three times for 5 min each with PBS. The sections were blocked with 3% normal horse serum in 0.3% TX-100 PBS for 20 min in room temperature, followed by incubation with 1:100 mouse monoclonal antibody to ß–amyloid, 1–16 (6E10) (Covance, NJ, United States) in 3% normal horse serum and 0.3% TX-100 PBS for 1 h at room temperature, and then overnight at 4°C. Non-specific isotype IgG1 (Sigma Aldrich) matching the species of primary antibody were used on negative control tissue sections. Next, the sections were washed in PBS, then incubated with a secondary antibody of biotinylated anti-mouse made in horse (MJS Biolynx, ON, Canada) at 1:100 for 45 min at RT followed by washing in PBS. The sections were then incubated in Vectastain Elite ABC HRP solution (MJS Biolynx, ON, Canada) for 30 min at RT then washed with PBS again. For visualization, the sections were developed using the Vector^®^ AEC substrate kit (MJS Biolynx, ON, Canada) and were counterstained with Mayer’s Hematoxylin (Sigma Aldrich) for nuclei. The sections were coverslipped with aqueous mounting medium for brightfield microscopy. The slides were stored at 4°C in a light tight box away from light. The brain tissue from an APP/PS1 transgenic mouse sacrificed at 19 months was used as a positive control tissue and processed along with experimental tissues (Ning et al., 2008). The Aβ immunohistochemistry on retinal cross-sections was used to confirm laminar distribution of immunolabeling observed in retinal wholemount preparations and to process controls such as omission of the primary antibody (to control against non-specific immunolabeling due to secondary antibody) and to assess for potential autofluorescence that may confound fluorescent imaging of the wholemount preparations (Supplementary Figure S3).

**FIGURE 1:**
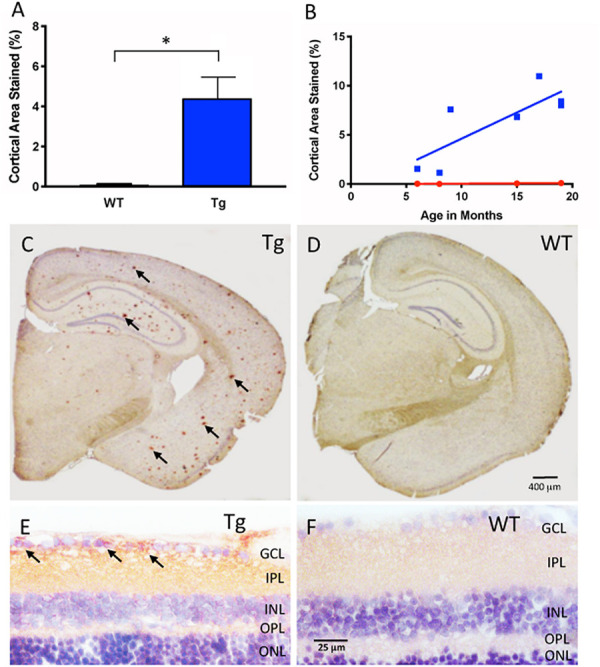
Aβ immunohistochemical cortical 6E10 immunostaining. **(A)** Tg mice (blue) had significantly higher percentage of cortical area immunostained relative to their WT controls (red) (Tg: 4.44% ± 1.50, *N* = 7; WT: 0.15% ± 0.08, *N* = 4; *p* < 0.05; Two-tailed Mann Whitney *U* test). **(B)** There was a trend towards higher cortical immunostaining in Tg mice (blue) that begins at nine months of age. There was a moderately strong correlation and increase of immunostaining over time for Tg mice (r^2^ = 0.639, slope = 0.53 ± 0.18, but not WT mice r^2^ = 0.85, slope = 0.006 ± 0.002; *p* = 0.77). **(C)** Representative cross-section of an 18 month Tg mouse brain immunoreacted for Aβ. Note the numerous Aβ plaques shown by the red (AEC) chromogenic reaction product in cortex and hippocampal areas (arrows), compared to **(D)**, the age-matched WT brain immunoreacted for Aβ. Scale bar for **(C,D)** is shown in *D* = 400 microns. **(E)** Retinal cross-section of an 18 month Tg mouse eye demonstrating Aβ immunoreactivity (arrows) in the inner retina in the ganglion cell layer (GCL) and the inner plexiform layer (IPL) compared to **(F)** the age-matched WT retina, which demonstrates a pale pink baseline level of immunoreactivity. Scale bar in *D* = 400 microns. Scale bar for **(E,F)** is shown in *F* = 25 microns. *Denotes significance.

A blind analysis was carried out on a series of immunoreacted Tg and WT brain sections from mice between the ages of 5 to 19 months. Images were taken on a brightfield Nikon Eclipse 80i (Nikon Corporation, Japan) to quantify the amount of Aβ immunostaining in the cortex of the brain. Figures 1C,D illustrates representative images of Tg and WT brain sections. A blinded quality control was conducted in order to discard images that were deemed of low quality, as a result of image acquisition or poor quality tissue processing due to tears in brain or retinal cross-sections.

The boundaries of the cortex assessed for plaques after Aβ immunohistochemistry included tissue from the corpus callosum (at the anteromedial visual area) to the end of the perirhinal area. Blood vessels and immunostaining artifacts were manually excluded from the analysis. For each image, the cortex was cropped using the magnetic lasso tool in Adobe Photoshop (Adobe Inc., San Jose, CA, United States) according to the outline specified by the Allen Mouse Brain Atlas (Allen Institute of Brain Science, 2004). Densiometric analysis was used to calculate the percentage of pixels of Aβ immunoreactivity compared to the total number of pixels in the cropped region of interest (ROI). Briefly, the ‘Color Range’ command in Adobe Photoshop was used to select a maximum range of wavelengths for all the positive labeled pixels within an ROI by an experienced rater. Next, the selected range was saved in an.axt file as the standard color range for positive immunolabeling. The.axt file was applied to all brain sample images, and a histogram depicting the number of pixels that fall within the standard color range within the ROI was generated, and recorded for analysis to compare between Tg and WT groups.

### *Ex vivo* Retinal Whole-Mount Aβ Immunofluorescence

Mouse eyecups were dissected and briefly soaked in solution of hyaluronidase type I-S solution (Sigma Aldrich, St. Louis, MO, United States) to liquefy and remove remaining vitreous. Under a dissecting microscope, fine forceps were used to carefully remove thin strands of vitreous before proceeding to the next step. The free floating wholemount retinal tissue then underwent antigen retrieval in 88% formic acid for 5 min at room temperature. The wholemount was washed 3 times in phosphate buffered saline (PBS, pH 7.4) and then underwent blocking with 3% normal goat serum diluted in 0.3% Triton-X (TX)-100-PBS solution to minimize nonspecific immunostaining. The wholemount was then incubated in 6E10 mouse monoclonal antibody, diluted in serum and PBS with 0.3% TX-100 at a working concentration of 1:100 for 1 h at room temperature and then overnight at 4°C. Negative controls were obtained by treating the wholemount with an irrelevant IgG1 isotype at the same concentration in place of the primary antibody incubation. After incubation in the primary antibody, the wholemount was thoroughly washed and incubated in fluorescent goat-anti-mousse Cy3 secondary antibodies (1:400) for 45 min at room temperature and rinsed 3 times in PBS, then followed by incubation in DAPI (1:500) for 10 min at room temperature for nuclear staining. Next, the wholemount was thoroughly washed 4 times at 15 min each on the shaker table to remove exogenous debris, and carefully placed on a glass slide with mounting medium and coverslipped. Confocal images were taken using a Zeiss 510 confocal microscope with Zen 2009 software (Carl Zeiss, Germany). Aβ clone 6E10 labeling by Cy3 was imaged with 543 nm excitation (false color red). Nuclear labeling by DAPI was imaged with 405 nm (false color blue). In order to assess autofluorescence of retinal wholemounts, and cross-sections, some tissues were processed without antibody incubations, but counterstained with DAPI and coverslipped before confocal microscopy. A blinded quality control was conducted where images that were deemed of low quality, as a result of image acquisition or artifactual labeling due to tears in retinal tissue during processing, were discarded prior to analysis.

**FIGURE 2:**
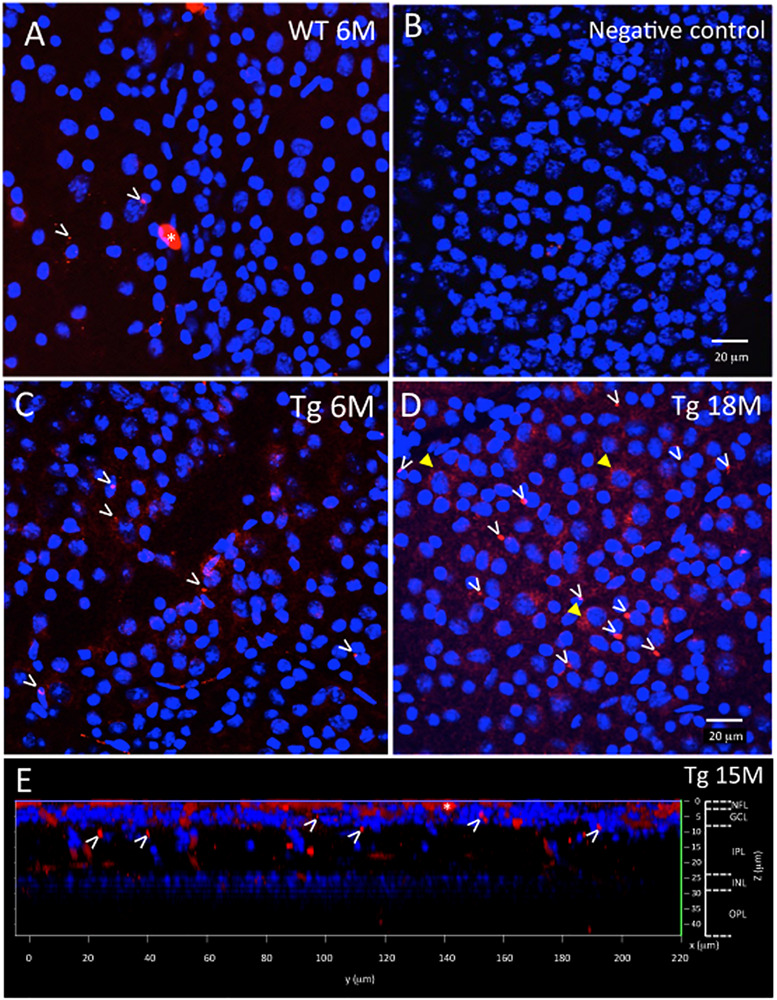
*Ex vivo* WT and Tg mouse wholemount and 6E10 immunofluorescence. Aβ deposits in the *ex vivo* retinal wholemount were identified by 6E10 immunofluorescence (red) and confocal imaging taken at the level of the GCL. The confocal imaging was initiated at the NFL, and as the optical steps proceeded deeper into the wholemount, DAPI (blue) nuclear staining was used to identify the beginning, middle and end of the GCL. All images in Figure 2 are shown at the level of the mid GCL. **(A)** Representative confocal image after Aβ immunofluorescence of a younger (6 month) WT retina. Note the small specks of Aβ deposits in red immunofluorescence, some shown by white arrowheads. A larger opaque red profile is an artifact (white asterisk). **(B)** Representative image of a negative control section, in which the primary antibody was replaced with a non-specific IgG. Image demonstrates very low background levels of red immunofluorescence. **(C)** Representative image of Aβ immunoreactivity in the 6 month Tg retina with representative red specks of immunofluorescence shown by white arrowheads. **(D)** In an older (18 month) Tg retina, the retinal immunofluorescence increased with more red immunoreactive specks (white arrowheads) and an occasional immunoreactive retinal ganglion cell (yellow arrowhead). **(A–D)** Scale bar: 20 μm. **(E)** An orthogonal view of the confocal z-stack reconstruction from a 15 month Tg retina demonstrates that the majority of the Aβ immunoreactivity is present in the inner retina, specifically in the NFL and GCL. Some Aβ immunoreactive specks can be seen extending deeper into the IPL. Cell nuclear labeling with DAPI (blue) reveals lamination in the retina. Lamination and scale bars are shown on the right axes.

Aβ load in the *ex vivo* retina was identified as bright immunofluorescence specks or intracellular labeling of retinal ganglion cells (RGC) in the confocal images manually identified by blinded raters (see examples of labeling in Figure 2). The number of bright immunofluorescent specks and intracellular labeled RGCs in each image were counted and normalized by the area of the retinal tissue in the image excluding any artifactual labeling caused by tears in tissues and labeling associated with blood cells within blood vessels, to yield a speck count per tissue area in mm^2^.

### *In vivo* Retinal Fluorescence Imaging With Curcumin

Mice underwent a lateral tail vein injection of the curcumin-based FluoroProbe that binds to Aβ. Approximately 2 h before eye imaging, animals were anesthetized by isoflurane gas and placed on a heating pad. Next, the animal’s tail was warmed in a container of lukewarm water to cause vasodilatation of the vein. The tail was then swabbed with alcohol, and one of the lateral veins was visualized. With the bevel of a 27-gauge needle facing upwards, the needle was inserted almost parallel to the vein, approximately 2 mm. After visualization of blood in the hub of the needle, 0.08 mL of a filter-sterilized curcumin solution in PBS (Sigma-Aldrich, St. Louis, MO, United States) was administered, for a final concentration of 0.75 mg/kg body weight.

To determine if curcumin injection in Tg mice would allow for better visualization of retinal fluorescence, we compared retinal fluorescence of Tg mice to their WT controls at multiple time points. Prior to each imaging session, the mouse was anesthetized with subcutaneous injection of the anesthetic cocktail containing ketamine at 100 mg/kg of body weight and dexmedetomidine at 0.1 mg/kg of body weight. Then, a drop of 1% Tropicamide was used on each eye to dilate the pupil. A zero-diopter contact lens was placed on the cornea to prevent dehydration and the mouse eyes were aligned to the system without any further contact. Each eye was aligned such that the optic nerve head was in the center of the field of view.

*In vivo* imaging was performed at each time point one day prior to curcumin injections, and then 2 h after curcumin injection. A custom SLO was designed for fluorescence imaging of the mouse retina. The SLO used 488 nm excitation light and broadband emission filters for the detection of fluorescence emissions longer than 500 nm. The imaging field of view was 50-degress or ∼1.7 mm across the retina. A tunable lens provided the ability to adjust the axial focal plane of the imaging system and the optimal focus on the retinal nerve fiber layer - ganglion cell layer was determined by the sharpness of the retinal blood vessels. Imaging frames were acquired at 5 frames per second and 50 frames were averaged to produce each image.

The amount of retinal fluorescence was measured quantitatively by automatically segmenting and counting the number of pixels in the fluorescent specks in each fSLO image. Intensity-based median filtering and thresholding was used to detect the specks. Blind quality assessment was performed and images with poor image quality (blurriness, noise) or segmentation errors (over or under-segmentation) were discarded from the final analysis.

### Statistics

Where indicated and appropriate, a two-tailed Mann-Whitney *U* test, a two-way ANOVA multiple comparison test with post hoc Bonferroni correction were conducted, and a linear regression model used. A finding of *p* < 0.05 was considered significant.

## Results

### Cortical Aβ Immunoreactivity Is Greater in Tg Than WT

Figure 1A shows cortical Aβ immunoreactivity in Tg and WT groups, and confirmed earlier studies of the APP/PS1 mouse model that demonstrates Aβ plaques in the Tg compared to the age-matched WT controls (Tg: 4.44% ± 1.50, *N* = 7; WT: 0.15% ± 0.08, *N* = 4, *p* < 0.05, two-tailed Mann Whitney U test). Immunohistochemistry demonstrated significant Aβ plaques in cortical layers and in hippocampus of the Tg compared to the WT brain (Figures 1C,D and Supplementary Figure S1). We then determined the age-related change in cortical Aβ load by using a linear regression model to compare immunoreactivity at different ages for both Tg and WT groups. Figure 1B demonstrates that Tg mice had an age-related increase in cortical labeling (r^2^ = 0.639, slope = 0.532 ± 0.179, *p* < 0.05), while WT mice did not (r^2^ = 0.851, slope = 0.00614 ± 0.0.00181, *p* = 0.77). Together, these data indicated that the Aβ immunoreactivity reliably detects the age-related increase in cortical Aβ load in the APP/PS1 mouse brain (Hsiao et al., 1996; Radde et al., 2006).

### Retinal Aβ Immunoreactivity Is Greater in Tg Than WT

Next, we used the same immunohistochemical methods for retina tissues. Retinal cross-sections and wholemounts processed for Aβ immunoreactivity demonstrated immunoreactivity with a different pattern from that observed in cortical brain areas. In the retinal cross-sections, the Aβ immunoreactivity resulted in a red (AEC) reaction product in the nerve fiber layer (NFL), ganglion cell layer (GCL) and inner plexiform layer (IPL) of the Tg eye with densely labeled areas surrounding retinal ganglion cells (arrows, Figure 1E). The age-matched WT eye revealed a light pink-red (AEC) background level of immunoreactivity in the NFL, GCL and IPL (Figure 1F). Deeper layers of the retina including the inner nuclear layer (INL), outer plexiform layer (OPL) and outer nuclear layer (ONL) were also assessed, but generally had less immunoreactivity than the inner retina.

**FIGURE 3:**
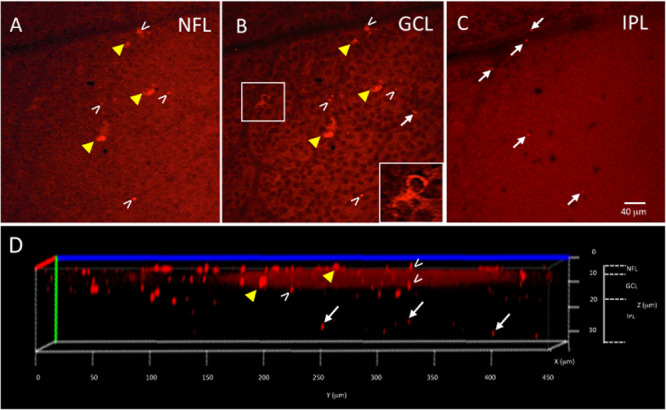
*Ex vivo* Tg mouse wholemount and 6E10 immunofluorescence. Aβ load in the *ex vivo* retina wholemount was identified by 6E10 immunofluorescence and z-stack confocal imaging taken through the NFL, GCL and IPL. **(A)** Representative high power confocal image of the NFL demonstrating red immunofluorescence. Note that Aβ specks can be seen in the NFL (white arrowheads), while the fluorescence associated with the RGC cell bodies in the GCL is also seen (yellow arrowheads). **(B)** As the confocal imaging descends into the wholemount, the GCL is identified in an optical slice by the presence of nuclei, here shown as black circular profiles as the DAPI channel is not shown. Note the fluorescence of the RGCs (yellow arrowheads) is evident in the cytoplasm (shown in boxed inset), and align with the RGCs seen in A. The extracellular Aβ specks can be seen (white arrowheads). **(C)** An optical slice through the IPL reveals fewer nuclei, as evidence by the lack of black circular nuclear profiles as seen in B. A few Aβ specks (white arrowheads) are seen, but generally less Aβ immunoreactivity is observed in IPL. Scale bar for **(A–C)** is shown in C = 40 microns. **(D)** The orthogonal view of the confocal z-stack reconstruction from the Tg retina shown in **(A–C)**. Note red immunoreactivity in the NFL, GCL and IPL (white arrowheads). The immunoreactivity present in the IPL (white arrows) indicates that the antibody penetrated deep into the wholemount tissue. Additional optical slices from this wholemount are shown in Supplementary Figure S2.

Figures 2, 3 show representative examples of immunofluorescence in the wholemount preparation. Wholemounts were imaged by confocal microscopy using z-stacks to collect data throughout the inner retina, including the NFL, GCL, IPL, and INL. Identification of retinal layers within the z-stacks was possible by observing the nuclear labeling with DAPI, present in the GCL and INL, but absent in NFL, IPL, OPL (Figure 2E and Supplementary Figure S2).

The wholemount retina revealed a pattern of Aβ speck-like deposits present in both Tg and WT mice. These speck-like deposits were sparsely distributed in the WT group of all ages. The wholemounts from younger Tg mice displayed low levels of Aβ speck-like deposits, but these became more numerous in the older Tg mice as shown by the white arrowheads in Figure 2. The wholemounts from older Tg mice also displayed Aβ labeled RGCs (Figures 2D, 3A,B yellow arrowheads). Confocal z-stack reconstructions of the retina wholemount images demonstrated that the majority of Aβ immunoreactivity was present in the inner retina, specifically in the NFL, GCL and IPL (Figures 2E, 3D and Supplementary Figure S2), consistent with the cross-sections shown in Figures 1E,F and in earlier findings in the APP/PS1 retina (Ning et al., 2008).

**FIGURE 4:**
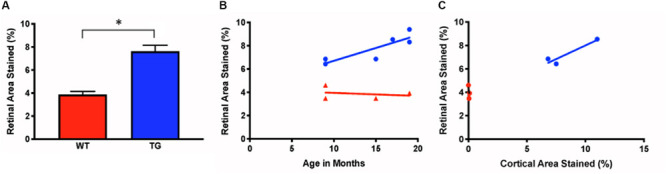
Measurements of *ex vivo* retinal Aβ immunofluorescence in Tg and WT retina. **(A)** *Ex vivo* retinal Aβ immunoreactivity was significantly higher in Tg (blue) than WT (red) (Tg: 7.63% ± 0.52, *N* = 6; WT: 3.87% ± 0.26, *N* = 4; *p* < 0.05; Two-tailed Mann Whitney *U* test). **(B)** Retinal Aβ increased with age in the Tg (blue), but less so in the WT (red) (r^2^ = 0.74, vs. r^2^ = 0.06, *p* = 0.76. **(C)** Cortical and retinal *ex vivo* labeling correlated better in Tg (blue) than WT (red) mice (Tg: r^2^ = 0.88, *N* = 3, *p* = 0.26; WT: r^2^ = 0.43, *N* = 3, *p* = 0.55). *Denotes significance.

The densiometric measurements of retinal Aβ immunoreactivity revealed that the Tg group had significantly greater retinal Aβ immunoreactivity than the WT controls (Tg: 7.63% ± 0.52, *N* = 6; WT: 3.87% ± 0.26, *N* = 4; *p* < 0.05, two-tailed Mann Whitney *U* test) (Figure 4A). Aβ increased with age in the Tg group, but less so in the WT group (r^2^ = 0.74, *p* < 0.05, vs. r^2^ = 0.06, *p* = 0.76) (Figure 4B).

Next, we assessed the relationship between the retinal and cortical Aβ immunoreactivity at different ages using a linear regression model for individual Tg and WT mice. There was a strong correlation for cortical and retinal labeling within each mouse for the Tg group, but not within the WT group (Tg: r^2^ = 0.88, *N* = 3, *p* = 0.26; WT: r^2^ = 0.43, *N* = 3, *p* = 0.55) (Figure 4C).

### Retinal *in vivo* Fluorescence After Curcumin Injection Is Higher in Tg Than WT Mice and Increases With Age

**FIGURE 5:**
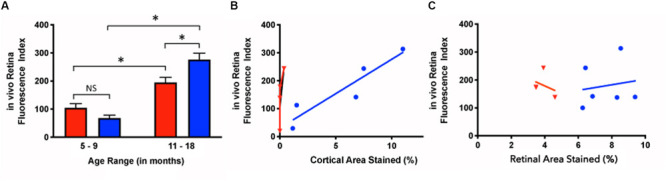
*In vivo* retinal fluorescence in Tg and WT retina in younger and older mice. **(A)** *In vivo* retinal fluorescence was similar between younger Tg (blue) and WT (red) mice (Tg: 68.1 ± 56.7, *N* = 18; WT: 104.7 ± 62.8, *N* = 8; *p* > 0.05). With increasing age, the amount of retinal fluorescence increased significantly for both Tg (younger Tg: 68.1 ± 56.7, *N* = 18; older Tg: 276.5 ± 108.6, *N* = 29; *p* < 0.05) and WT groups (younger WT: 104.7 ± 62.8, *N* = 8; older WT: 194.8 ± 52.5, *N* = 22; *p* < 0.05). However, within the older group (11–18 months), the Tg mice had significantly greater retinal fluorescence than the WT mice (Tg: 276.5 ± 108.6, *N* = 29; WT: 194.8 ± 52.5, *N* = 22; *p* < 0.05) Statistics in A was undertaken with a two-way ANOVA multiple comparisons with post hoc Bonferroni correction. **(B)** The correlation between *in vivo* retinal fluorescence and *ex vivo* cortical immunoreactivity was stronger in Tg (blue) compared to WT (red) mice (Tg: r^2^ = 0.859, slope = 24.6 ± 5.75, *N* = 5, *p* < 0.05; WT: r^2^ = 0.522, slope = 370 ± 250, *N* = 4, *p* = 0.28). **(C)** When comparing *in vivo* fluorescence and *ex vivo* retinal immunolabeling, Tg (blue) had a weak positive correlation (r^2^ = 0.0251, slope = 10.0 ± 31.1, *N* = 6, *p* = 0.76) while WT (red) had a weak negative correlation (r^2^ = 0.107, slope = -27.1 ± 55.3, *N* = 3, *p* = 0.67). *Denotes significance.

Younger mice displayed retinal fluorescence after curcumin injections in both the Tg and WT groups (Figure 5A). First, there was no significance between the *in vivo* retinal fluorescence levels of the Tg compared to the WT mice in the younger group (5–9 months) (Tg: 68.1 ± 56.7, *N* = 18*;* WT: 104.7 ± 62.8, *N* = 8; *p* > 0.05). However, with increasing age, the amount of retinal fluorescence increased significantly for the Tg group (younger Tg: 68.1 ± 56.7, *N* = 18; older Tg: 276.5 ± 108.6, *N* = 29; *p* < 0.05) as well as in the WT group (younger WT: 104.7 ± 62.8, *N* = 8; older WT: 194.8 ± 52.5, *N* = 22; *p* < 0.05). Furthermore, within the older group (11–18 months), the Tg mice had significantly greater retinal fluorescence than the WT mice (older Tg: 276.5 ± 108.6, *N* = 29; older WT: 194.8 ± 52.5, *N* = 22; *p* < 0.05; two-way ANOVA multiple comparison test with post hoc Bonferroni).

### Retinal *in vivo* Fluorescence Correlates With *ex vivo* Cortical Aβ Loads

**FIGURE 6:**
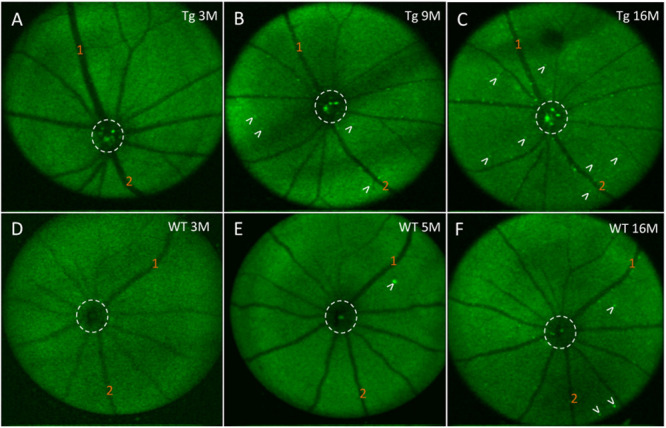
*In vivo* fluorescence of the mouse retina after curcumin tail vein injections. **(A–C)** *In vivo* longitudinal fluorescence images of an individual Tg mouse at 3, 9, and 16 months of age. **(D–F)** *In vivo* longitudinal fluorescence images of an individual WT mouse at 3, 5, and 16 months of age. The pattern of Aβ *in vivo* fluorescence is seen as bright green specks (white arrowheads). Note that the Aβ *in vivo* fluorescent deposits increased as the animal aged. Representative landmark blood vessels are labeled with orange numbers and can be identified in each of the images in the Tg **(A–C)** and in the WT **(D–F)**. Dashed white circle indicates area of the optic nerve head. Representative examples of the *in vivo* longitudinal fluorescence images of an individual WT mouse at 3, 5, and 16 months of age reveals that Aβ fluorescent deposits only marginally increased as the animal aged **(D–F)**. Note a significantly higher load of Aβ fluorescent deposits in the 16 month Tg **(C)** compared to the 16 month WT **(F)** mice.

Next, we assessed the relationship between *in vivo* retinal fluorescence associated with curcumin injections and *ex vivo* cortical Aβ loads assessed with Aβ immunoreactivity. *In vivo* fSLO images are shown for a representative Tg mouse at 3, 9, and 16 months old (Figures 6A–C) compared to *in vivo* images from a WT mouse at 3, 5, and 16 months old (Figures 6D–F). Note the increased levels of *in vivo* fluorescent “specks” (white arrowheads) in the Tg compared to WT retinal images. A linear regression model was used to identify the correlation between the *in vivo* retinal fluorescence and *ex vivo* cortical immunoreactivity. Retinal *in vivo* fluorescence correlated stronger with *ex vivo* cortical Aβ immunoreactivity in Tg compared to WT mice (Tg: r^2^ = 0.859, slope = 24.6 ± 5.75, *N* = 5, *p* < 0.05; WT: r^2^ = 0.522, slope = 370 ± 250, *N* = 4, *p* = 0.28) (Figure 5B). There was weak positive, and weak negative correlation between *in vivo* and *ex vivo* retinal Aβ immunoreactivity, for Tg and WT mice, respectively (Tg: r^2^ = 0.0251, slope = 10.0 ± 31.1, *N* = 6, *p* = 0.76; WT: r^2^ = 0.107, slope = -27.1 ± 55.3, *N* = 3, *p* = 0.67) (Figure 5C).

## Discussion

### Amyloid-β and Age-Related Cortical Changes

The amyloid cascade hypothesis states that Aβ accumulation, via post-translational proteolytic cleavage of amyloid beta peptides (APP), precedes aggregation into senile plaques and subsequent neuronal damage (Beyreuther and Masters, 1991; Hardy and Allsop, 1991; Selkoe, 1991; Hardy and Higgins, 1992). Whether Aβ, APP, or senile plaque accumulation promotes AD, or is a consequence of the disease, remains controversial, but histological evidence of Aβ and senile plaques is a hallmark of AD diagnosis and an increased burden of Aβ plaques promotes neurocognitive limitations in AD mouse models (Chen et al., 2000; Janus et al., 2000).

In this study, we used the APP/PS1 transgenic mouse model as it has been used previously to evaluate the process of amyloidogenesis at the pre-dementia phase of AD through the accumulation of Aβ deposits and plaques in the Tg mouse brain tissues and the retina (Ning et al., 2008; Zahs and Ashe, 2010; Shah et al., 2017). Using an Aβ antibody (clone 6E10), which recognizes the amino acid residues 1-16 of Aβ, we found greater cortical immunostaining in Tg relative to the WT controls – a difference that progressively increased over time, which has been shown by others (Radde et al., 2006; Maia et al., 2013; Georgevsky et al., 2019). This consistency with the literature both confirmed the expected phenotypical differences in our Tg and WT brains, and reassured us that the Aβ immunohistochemistry would detect Aβ deposits in the retina. This longitudinal study identified an age-related correlation of cortical Aβ, detected by immunohistochemistry, in the APP/PS1 transgenic mouse. More importantly, our study demonstrated a correlation between Aβ immunohistochemistry in the cortex and *in vivo* fSLO retinal imaging after curcumin injections (Figure 5B).

### Retinal Fluorescence After Tail-Vein Injections of Curcumin

Koronyo-Hamaoui et al. (2011) showed high-resolution and specific visualization of retinal Aβ plaques using fluorescent dye (curcumin) injections and fSLO retinal imaging of live APP/PS1 mice. Curcumin is a natural product and a non-toxic fluorochrome that binds to Aβ plaques with no significant side effects found in mice or humans (Yang et al., 2005; Garcia-Alloza et al., 2007; Baum et al., 2008). Using a modified protocol of Koronyo-Hamaoui et al., we imaged mice every 2 months over an 18-month period. Interestingly, we found no difference in *in vivo* retinal fluorescence between Tg and WT mice in the younger mice (5–9 months), yet significant differences between the two cohorts in the older mice (11–18 months). We found *in vivo* retinal fluorescence in both the WT and Tg mice, and this suggests that even in the WT retina it is possible that Aβ deposits (bound to curcumin) and/or autofluorescent components are present in the WT retina. We also found an age-related increase in *in vivo* retinal fluorescence in the WT group (Figure 5A) which suggests that even in WT mice, Aβ increases with age, consistent with a previous study which demonstrated an age-related increases in the retinal Aβ in control C57BL/6J mice and normal (non-AD) postmortem human eyes assessed by immunohistochemistry (Kam et al., 2010). Furthermore, our *ex-vivo* immunohistochemistry suggests that the retinal fluorescence is associated with Aβ immunoreactivity, as low levels of Aβ immunoreactivity were present in WT cross-sections (Figure 1F) and in the WT retinal wholemounts (Figures 2A, 4A).

Another possibility is that the retinal fluorescence observed here is associated with autofluorescent changes due to aging. Such changes have been reported as subretinal microglia containing autofluorescent lipofuscin granules that migrate from the inner retina to the subretinal space (Xu et al., 2008). While this is a possibility, it is less likely because microglial profiles were not observed in any of our *in vivo* fluorescent images or in the *ex vivo* Aβ immunohistochemistry. Also, the *in vivo* fluorescent images were taken at the level of the NFL and GCL, far from the RPE layer where the majority of microglia (with autofluorescent lipofuscin granules) reside. However, in a recent study by Harper et al., microglia cells in the inner retina were observed after Aβ immunohistochemistry in an *ex vivo* wholemount retina suggesting that a component of the *in vivo* retinal fluorescence may be from microglia in the inner retina in the APP/PS1 retina (Harper et al., 2020).

Despite the age-related increases in both WT and Tg *in vivo* retinal fluorescence, the Tg mice have significantly higher retinal fluorescence in the older age group, suggesting that this difference cannot be attributed to autofluorescence alone, and likely involves additional Aβ deposition in the Tg (but not the WT) retina. Since the APP/PS1 mutation is what differentiates the Tg and WT mice, the increased retinal fluorescence in older Tg mice is likely due to impaired Aβ metabolism and deposition associated with the transgenes, which may lead to retinal structural and functional changes associated with this transgenic model.

### Does the APP/PS1 Mouse Model Display Retinal Functional or Structural Changes With Age?

It is possible in this mouse model that the impaired Aβ metabolism leads to retinal structural and functional changes, which may also delineate useful differences associated with the AD eye. Several non-invasive *in vivo* imaging techniques have shown retinal changes in the AD patient (for review see Hampel et al., 2018). These include, spectral domain optical coherence tomography, and electroretinograms (ERG) (Javaid et al., 2016; Snyder et al., 2016; van Wijngaarden et al., 2017) and scanning laser ophthalmoscopy (Janus et al., 2000). Being able to observe structural or functional changes in the AD eye would eliminate the necessity to label retinal Aβ with Fluoroprobes such as curcumin, a step that requires systemic administration of the Fluoroprobe. The APP/PS1 mouse has been used to identify structural changes that may precede cortical changes. Georgevsky et al. measured ERGs and found a functional change in the b-wave of the ERG beginning at 3 months of age, which was significantly earlier than retinal structural changes and thinning of the inner retina (between the ganglion cell layer and the inner nuclear layer). Interestingly, the structural changes they observed at 9 months of age in the APP/PS1 model were in the inner retina, which is the location of the enhanced *in vivo* fluorescence and Aβ immunolabeling we found in our studies (Georgevsky et al., 2019). In another study, Harper et al. used multi-contrast OCT on the APP/PS1 model to obtain a combination of imaging data on standard reflectivity, polarization-sensitive OCT and OCT angiography, but concluded that there were few retinal structural differences between the APP/PS1 and WT controls (Harper et al., 2020). Nevertheless, these studies on *in vivo* multimodal imaging of the eye are encouraging as it is likely that future work will require a combination of methods to assess retinal functional and structural changes, as well as labeling AD biomarker peptides Aβ (and/or tau) to track the onset and progression of AD in animal models and later in the AD patient population.

### Limitations of the Study

Interestingly, we found limited correlation between the measurements of retinal *in vivo* retinal fluorescence and *ex vivo* Aβ immunoreactivity in wholemount tissues in both mouse cohorts. This was unexpected, and likely associated with challenges in undertaking immunohistochemistry in retinal wholemount tissues. The fragility of the mouse retinal tissue and the need to process free-floating wholemount preparations can lead to artifactual tears and folds in tissue during processing. Aberrations and artifactual tears limited our ability to obtain data on all *ex vivo* retinas. Harper et al. also found limitations in quantifying Aβ immunohistochemistry in retinal wholemounts and suggested that in their study a strong immunofluorescent signal appeared to be derived from a range of sources, many of which were non-specific for Aβ (Harper et al., 2020). We confirmed by z-stack image projections that the immunofluorescence in the *ex vivo* wholemounts came from the inner retina (GCL) and not the vitreous interface (Figures 2E, 3D). While we also confirmed a lack of autofluorescence due to microglia in retinal cross sections (Supplementary Figures S3A–D), it is still possible that these limitations may have contributed to the relatively weak correlation we found in comparing our fluorescent *in vivo* and *ex vivo* retinal results (Figure 4C).

Another limitation of the study is the use of mouse models. While the APP/PS1 is a well-established AD model, it is a genetically engineered mouse model with inherent differences from human AD. Transgenic mouse models incorporate a variety of promoters to overexpress familial AD-associated mutations in APP and PS1, potentially leading to variability in Aβ expression, which may partially account for the inter-mouse variability in our results (Chang and Suh, 2005; Mitani et al., 2012; Nicolas and Hassan, 2014; Kerridge et al., 2015; Nhan et al., 2015; Willem et al., 2015). Moreover, overexpression of non-Aβ-producing APP fragments could lead to non-physiological interactions with endogenous proteins, potentially adding further to the variability within and amongst the many transgenic models used for AD studies (Jucker and Walker, 2013). Finally, there are intrinsic differences (and similarities) in the interconnections between the brain and eye of the mouse and human, which make mouse models comparable, but not identical, to human. Together, these limitations make it difficult to directly translate our findings to humans. Further studies are required to build on this proof-of-concept, with the intention of eventual clinical application.

### Correlation of *in vivo* Retinal Fluorescence to Cortical Aβ

*In vivo* optical imaging of retinal Aβ plaques after curcumin injection has been done with high resolution and specificity for review, see Koronyo-Hamaoui et al. (2011), Goozee et al. (2016) yet ours is the first to correlate these findings with cortical Aβ load. We observed a correlation between *in vivo* retinal fluorescence and *ex vivo* cortical Aβ immunoreactivity in Tg mice. Since cortical Aβ load predicts AD progression, the correlation we found between our *in vivo* retinal fluorescence and the *ex vivo* cortical data suggests that *in vivo* retinal fluorescence imaging may be useful for determining cortical Aβ load.

Determining if we could detect retinal changes that reflect or precede cortical abnormalities in the APP/PS1 mouse model was one of the primary motivations underlying this longitudinal study. Our data from *ex vivo* immunohistochemistry and *in vivo* curcumin fluorescence measurements consistently demonstrated that 9 months of age is approximately the age when APP/PS1 Tg mice show significantly higher cortical and retinal immunoreactivity and *in vivo* retinal fluorescence after curcumin injections compared to WT (Figure 5A). Others have found Aβ-like accumulation in transgenic AD models to develop in pre-symptomatic stages, as early as 2.5 months, preceding their detection in the brain, and this may be related differences in the AD mouse models studied (Koronyo-Hamaoui et al., 2011; Hart et al., 2016). Retinal fluorescence at such a young age may be secondary from background autofluorescence or due to non-specific labeling of the Aβ antibody as suggested by others (Xu et al., 2008; Harper et al., 2020).

Using the same strain as employed here, Georgevsky et al. showed retinal structural changes in both WT and Tg mice, but with enhanced changes in Tg mice after 9 months of age (Georgevsky et al., 2019), which is consistent with our results of a significant difference in (1) cortical and retinal Aβ immunoreactivity (Figure 4C), and (2) cortical Aβ immunoreactivity and *in vivo* retinal fluorescence after 9 months of age (Figure 5B). Our findings suggest that *in vivo* fSLO imaging of curcumin-injected mice may show enhanced retinal fluorescence that is in parallel to cortical Aβ deposition. Future work with improved curcumin derivatives that demonstrate higher fluorescence when bound to Aβ will have the potential to advance these studies towards the development of a non-invasive, *in vivo* method to image retinal Aβ as a surrogate method to assess Aβ load in the CNS.

## Data Availability Statement

The raw data supporting the conclusions of this article will be made available by the authors upon appropriate request.

## Ethics Statement

This study used APP/PS1 transgenic and non-transgenic control mice. The animal study was reviewed and approved by the UBC and SFU Animal Care Committees.

## Author Contributions

AS, SL, and JM wrote the manuscript, analyzed and interpreted the data, and generated the figures. DW and JC undertook the animal studies, curcumin injections, *in vivo* retinal imaging, and data collection. EleT, EllT, and JC processed the *ex vivo* retinal wholemounts and brain tissues, undertook microscopy/confocal imaging, and collected the data. EleT assisted in figure and manuscript preparation. AS and SL completed statistical tests. SL wrote data analysis algorithms and code. DM provided expert opinion and technical advice on analysis. JM, MS, and MB conceived and designed the study, obtained funding, interpreted the data, and critically revised the manuscript.

## Conflict of Interest

The authors declare that the research was conducted in the absence of any commercial or financial relationships that could be construed as a potential conflict of interest.

## Supplementary Material

FIGURE S1 Aβ immunoreactivity in Tg and WT brain tissues using chromogenic method and AEC (red) reaction product. **(A)** Brain of a 17 month Tg mouse demonstrated Aβ plaques throughout cortex and representative plaques identified by arrows in enlargement in **(B)**. **(C)** Brain of a 19 month WT had no plaques, but some small Aβ immunoreactive deposits shown at higher power in **(D)** (arrows). Tissues were counterstained with Hematoxylin (purple) for nuclei. Scale bar for B and D are shown in D = 50 microns.

FIGURE S2 Primary antibody penetration into the deeper layers of the retinal wholemount is confirmed by confocal z-stack reconstructions. **(A-F)** Sequential optical images are shown from a z-stack taken from a 9 month Tg wholemount after immunofluorescence using an antibody against Aβ. Both speck-like Aβ immunofluorescence (white arrowheads) and cytoplasmic labeling of RGCs (yellow arrowheads) are seen. As the DAPI (blue) channel is not shown here, the lamination of the retina can be confirmed by the presence of round black areas, which represent nuclei within the GCL, INL and ONL layers. Note some speck-like labeling is evident as deep as the OPL. Scale bar = 40 microns.

FIGURE S3 Control sections processed to identify autofluorescence in Tg and WT retina in cross-sections and wholemount retina. To assess for autofluorescence several cross sections were treated by standard deparaffinization, antigen retrieval and DAPI staining before coverslipping and confocal microscopy. Specifically, all steps for immunofluorescence were omitted. Cross-sections from a 9 month Tg retina **(A)** and 15 month Tg retina **(B)** were imaged under 563 nm (Cy3) and 405 nm (DAPI). Note lack of red fluorescent signal indicating no autofluorescence under 563 nm. A cross section of a 12 month WT retina **(C)** and a wholemount from a 12 month Tg retina **(D**) were imaged under 488 nm (FITC) and 405 nm (DAPI) again demonstrating lack of green fluorescent signal indicating no autofluorescence under 488 nm. **(E,F)** To assess the possibility of non-specific signals associated with immunohistochemistry, additional cross sections from a 12 month Tg retina **(E)** and a 15 month Tg retina **(F)**, were processed for immunohistochemistry, but with the omission of the primary antibody. Note lack of the AEC (red) immunoreactive product on both cross-sections counterstained with Hematoxylin (purple) for nuclei. Also note the presence of pigmented cells (arrows) on the NFL **(E)** and under the ONL **(F)** which may represent migration of melanin associated with a microglia or RPE cell (arrows).
