# Supplementary figures and images for: In vivo Retinal Fluorescence Imaging With Curcumin in an Alzheimer Mouse Model
Source: Front Neurosci. 2020 Jul 3;14:713. doi: 10.3389/fnins.2020.00713 (PMC7350785; doi:10.3389/fnins.2020.00713)

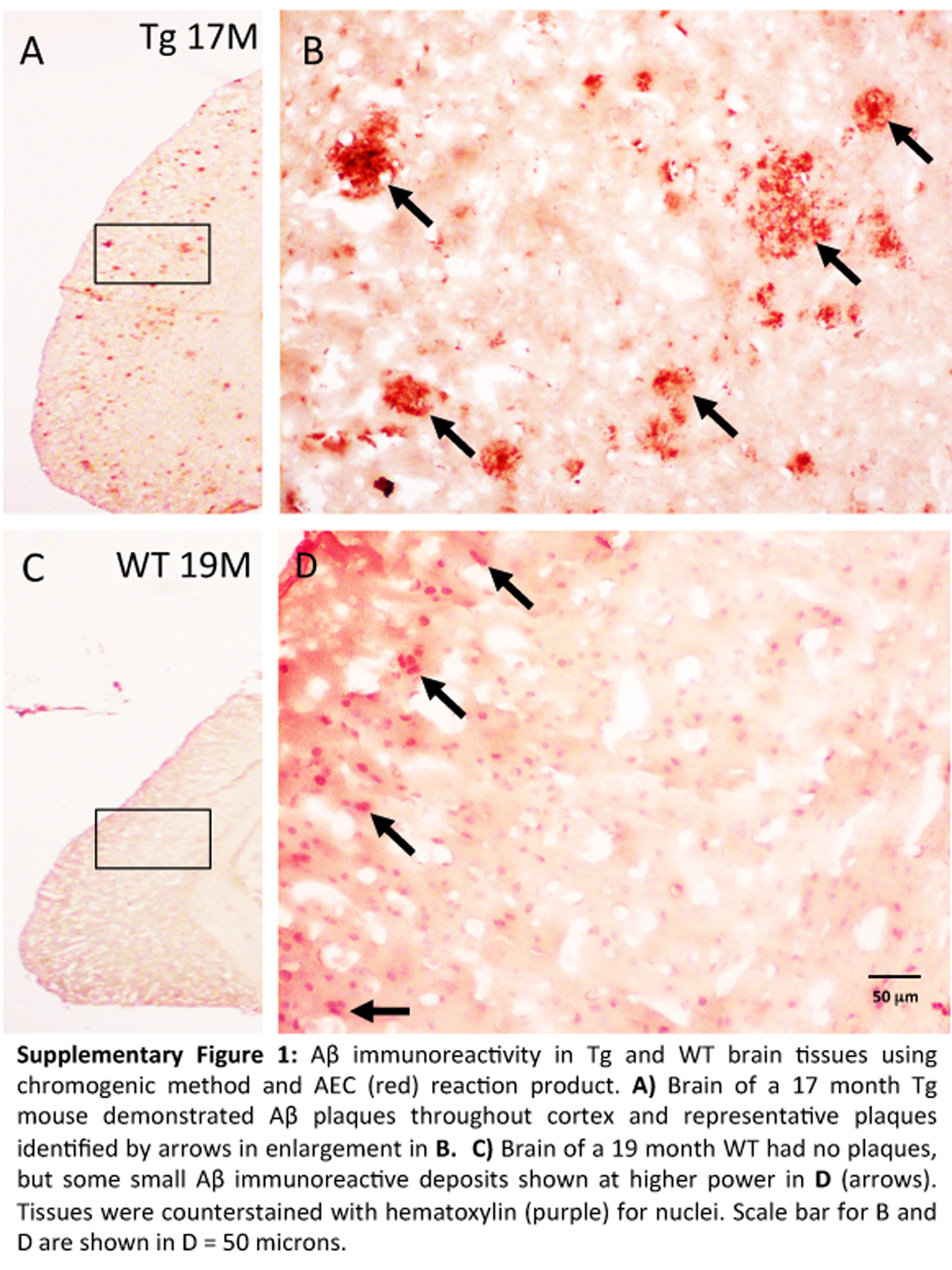

Supplement: FIGURE S1 — Aβ immunoreactivity in Tg and WT brain tissues using chromogenic method and AEC (red) reaction product. (A) Brain of a 17 month Tg mouse demonstrated Aβ plaques throughout cortex and representative plaques identified by arrows in enlargement in (B). (C) Brain of a 19 month WT had no plaques, but some small Aβ immunoreactive deposits shown at higher power in (D) (arrows). Tissues were counterstained with Hematoxylin (purple) for nuclei. Scale bar for B and D are shown in D = 50 microns. [file Image_1.TIF]

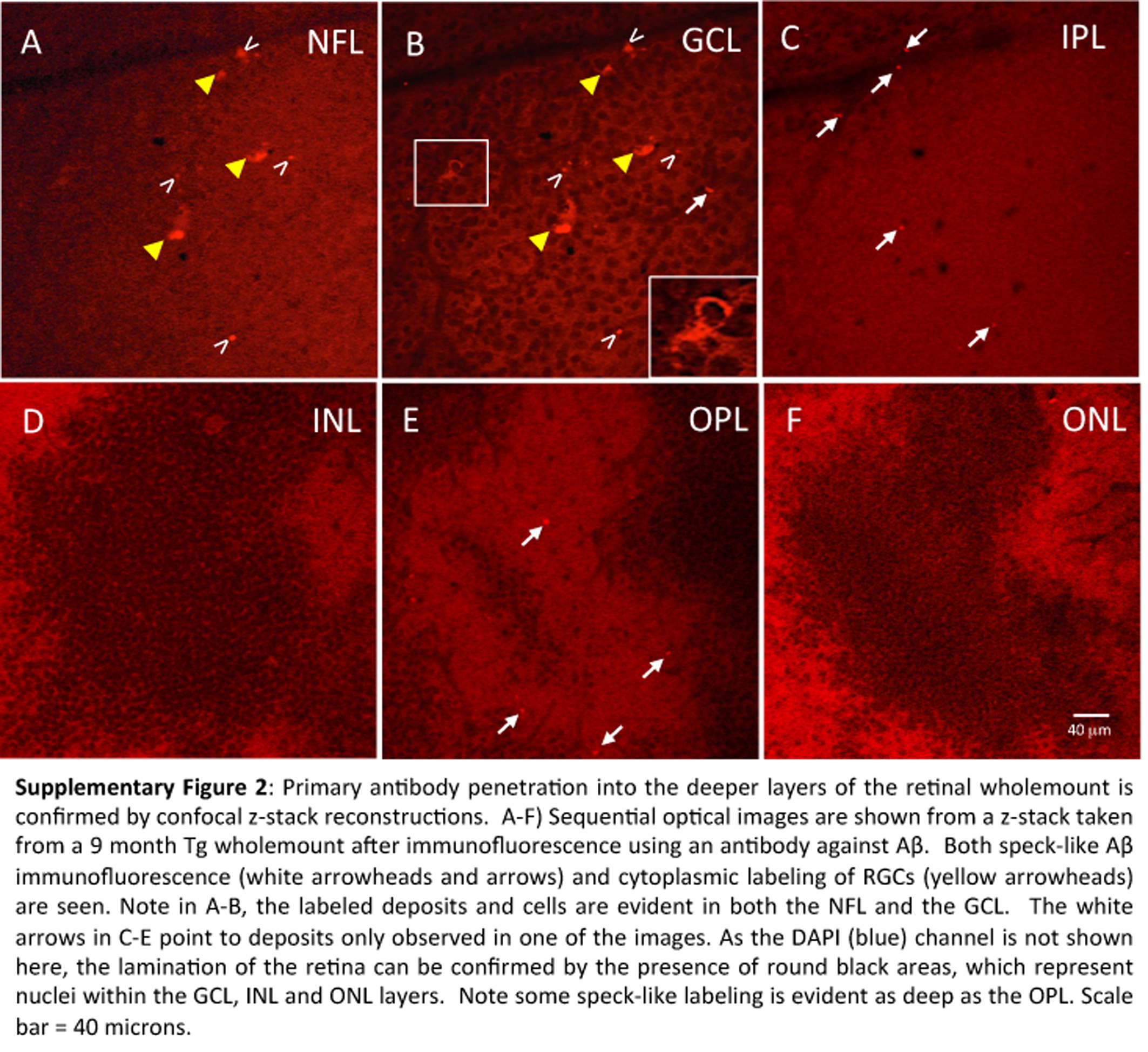

Supplement: FIGURE S2 — Primary antibody penetration into the deeper layers of the retinal wholemount is confirmed by confocal z-stack reconstructions. (A-F) Sequential optical images are shown from a z-stack taken from a 9 month Tg wholemount after immunofluorescence using an antibody against Aβ. Both speck-like Aβ immunofluorescence (white arrowheads) and cytoplasmic labeling of RGCs (yellow arrowheads) are seen. As the DAPI (blue) channel is not shown here, the lamination of the retina can be confirmed by the presence of round black areas, which represent nuclei within the GCL, INL and ONL layers. Note some speck-like labeling is evident as deep as the OPL. Scale bar = 40 microns. [file Image_2.TIF]

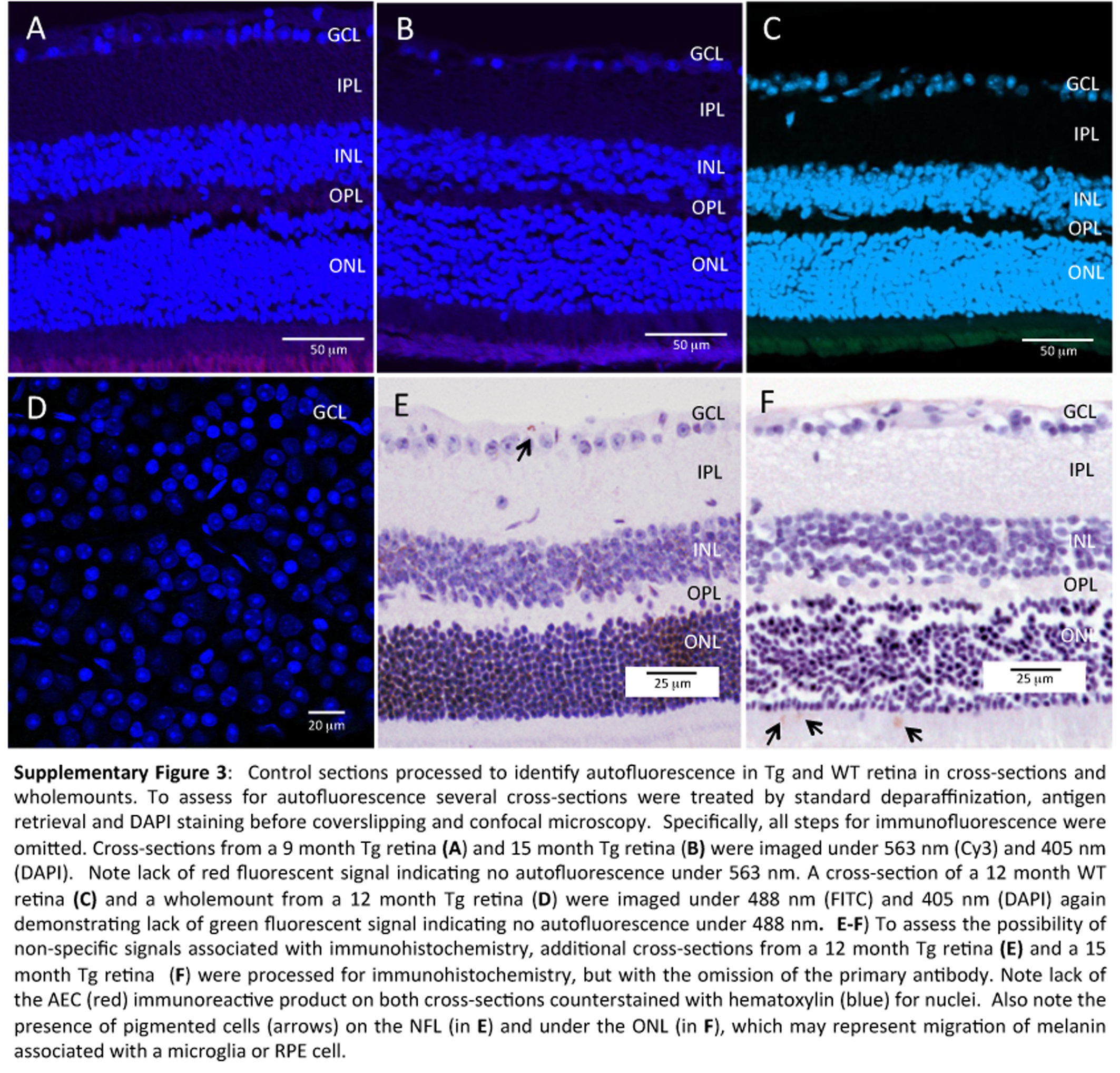

Supplement: FIGURE S3 — Control sections processed to identify autofluorescence in Tg and WT retina in cross-sections and wholemount retina. To assess for autofluorescence several cross sections were treated by standard deparaffinization, antigen retrieval and DAPI staining before coverslipping and confocal microscopy. Specifically, all steps for immunofluorescence were omitted. Cross-sections from a 9 month Tg retina (A) and 15 month Tg retina (B) were imaged under 563 nm (Cy3) and 405 nm (DAPI). Note lack of red fluorescent signal indicating no autofluorescence under 563 nm. A cross section of a 12 month WT retina (C) and a wholemount from a 12 month Tg retina (D) were imaged under 488 nm (FITC) and 405 nm (DAPI) again demonstrating lack of green fluorescent signal indicating no autofluorescence under 488 nm. (E,F) To assess the possibility of non-specific signals associated with immunohistochemistry, additional cross sections from a 12 month Tg retina (E) and a 15 month Tg retina (F), were processed for immunohistochemistry, but with the omission of the primary antibody. Note lack of the AEC (red) immunoreactive product on both cross-sections counterstained with Hematoxylin (purple) for nuclei. Also note the presence of pigmented cells (arrows) on the NFL (E) and under the ONL (F) which may represent migration of melanin associated with a microglia or RPE cell (arrows). [file Image_3.TIF]
